# Retraction: Design and synthesis of magnetic Fe_3_O_4_@NFC-ImSalophCu nanocatalyst based on cellulose nanofibers as a new and highly efficient, reusable, stable and green catalyst for the synthesis of 1,2,3-triazoles

**DOI:** 10.1039/d3ra90128a

**Published:** 2024-01-05

**Authors:** Pouya Ghamari kargar, Ghodsieh Bagherzade, Hossein Eshghi

**Affiliations:** a Department of Chemistry, Faculty of Sciences, University of Birjand Birjand 97175-615 Iran gbagherzade@gmail.com bagherzadeh@birjand.ac.ir +98 56 32345192 +98 56 32345192; b Department of Chemistry, Faculty of Science, Ferdowsi University of Mashhad Mashhad Iran

## Abstract

Retraction of ‘Design and synthesis of magnetic Fe_3_O_4_@NFC-ImSalophCu nanocatalyst based on cellulose nanofibers as a new and highly efficient, reusable, stable and green catalyst for the synthesis of 1,2,3-triazoles’ by Pouya Ghamari kargar *et al.*, *RSC Adv.*, 2020, **10**, 32927–32937, https://doi.org/10.1039/D0RA06251K

The Royal Society of Chemistry, with the agreement of the named author, hereby wholly retracts this *RSC Advances* article due to concerns with the reliability of the data.

The XRD patterns in Fig 4a–c and 10b contain repeating sections. The authors provided raw data for Fig. 4, but this was found to contain duplicated sections of data across different datasets representing different samples.

The raw XRD data provided by the authors for Fe_3_O_4_ in Fig. 4a of this article was identical in a number of different regions to the raw XRD data provided by the authors for CuO in Fig. 4b of this article and Fig. 3 of ref. 1. In Fig. 4c, the XRD data for Fe_3_O_4_@NFC-ImSalphCu was also found to contain identical sections of data within the raw dataset.

The authors have stated that they outsourced the XRD data collection to an external company.

Given the significance of these concerns, the findings presented in this paper are no longer reliable.

The authors were informed about the retraction of the article. Pouya Ghamari Kargar and Ghodsieh Bagherzade have not agreed with the decision.

Signed: Hossein Eshghi

Date: 13th December 2023

Retraction endorsed by Laura Fisher, Executive Editor, *RSC Advances*.

## Supplementary Material
